# An empirical investigation of the associations between metacognition, mindfulness experiential avoidance, depression, and anxiety

**DOI:** 10.1186/s40359-023-01336-7

**Published:** 2023-09-21

**Authors:** Torstein Ådnøy, Stian Solem, Roger Hagen, Audun Havnen

**Affiliations:** 1 Department of Psychology, Norwegian University of Science and Technology, Trondheim, Norway; 2 Department of Psychology, University of Oslo, Oslo, Norway; 3 Research institute, Modum Bad, Vikersund, Norway; 4 Division of Psychiatry, Nidaros Community Mental Health Centre, St. Olavs University Hospital, Trondheim, Norway

**Keywords:** Metacognition, Mindfulness, Experiential avoidance, Anxiety, Depression

## Abstract

**Background:**

The aims of this study were to explore the possible relation between metacognition, mindfulness, and experiential avoidance, as well as their association with symptoms of anxiety and depression.

**Methods:**

Cross-sectional data was collected from a community sample (*N* = 364) who completed the Metacognitions Questionnaire-30 (MCQ-30), the Five Facet Mindfulness Questionnaire-24 (FFMQ-24), the Acceptance and Action Questionnaire-II (AAQ-II), the Generalized Anxiety Disorder 7-item (GAD-7), and the Patient Health Questionnaire-9 (PHQ-9).

**Results:**

There were moderate-strong associations between mindfulness (FFMQ-24), metacognition (MCQ-30), and experiential avoidance (AAQ-II) (0.62 − 0.67), and they showed similar relations with symptoms of depression (PHQ-9) and anxiety (GAD-7) (0.57 − 0.71). Metacognition, experiential avoidance, and the non-judging subscale of FFMQ-24 constituted a latent factor of flexibility in cognition and emotional experience, while three FFMQ-24 subscales (describing, acting with awareness, and observing) constituted a present-centered attention and awareness factor. Regression analyses indicated that flexibility explained more of the variance in symptoms of anxiety and depression than present-centered attention and awareness.

**Conclusions:**

The results suggest that flexibility in cognitive and emotional regulation skills could be important in explaining symptoms of anxiety and depression.

## Background

The development of cognitive behavior therapy (CBT) could be divided into three waves [1]. The first wave was behavior therapy (BT) where behavioral principles like classical conditioning and operant learning were used in a clinical setting to treat psychological problems (e.g., phobias). The second wave was cognitive therapy (CT) developed by A.T. Beck in the early 1970s, which had a strong focus on information processing and content-oriented cognitive change [2]. From the late 1980s to early 1990s, there occurred a synthesis between behavior therapy and cognitive therapy, into cognitive behavior therapy. CBT today has a large evidence base supporting its efficacy across many conditions, populations and contexts [3]. During the last two decades, new treatments grounded in cognitive therapy have evolved, which many have classified as the ‘third wave’ of CBT [4]. Treatments in this category often focus on concepts such as metacognition, mindfulness, cognitive fusion, and experiential acceptance [2].

A somewhat recent type of cognitive therapy is metacognitive therapy (MCT). An important part of this therapeutic approach is the attempt to change patients’ metacognitive beliefs [5]. Metacognitions involve the psychological structures, knowledge, events, and processes that are involved in the control, modification, and interpretation of thinking itself [6]. According to the metacognitive model, mental disorder is caused by a maladaptive response style called the cognitive attentional syndrome (CAS) [5]. The CAS is characterized by extended thinking in the form of worry, rumination, fixated attention on perceived threats, and unhelpful coping behaviors such as suppressing thoughts, which leads to the development and maintenance of mental disorder. The activation and continuation of the CAS depends upon maladaptive metacognitive beliefs. A negative meta-belief could be that thoughts are dangerous, and that worry/rumination is uncontrollable, while an example of a positive meta-belief is that worry is helpful to prepare for possible threats. The Metacognitions Questionnaire (MCQ) [6] was developed to assess metacognitive beliefs.

Mindfulness, on the other hand, is a skill or a state of mind, consisting of a conscious focus on internal and external experiences in the moment [7], and is strongly associated with concepts like attention and awareness. Mindfulness is historically rooted in Buddhism, but parallels have been drawn to ideas from various other philosophical and psychological traditions [8]. It has been defined as “the awareness that emerges through paying attention on purpose, in the present moment, and nonjudgmentally to the unfolding of experience moment by moment.” [9, p. 145]. Moreover, non-reactivity to inner experience, non-judgmental awareness, and acceptance [10, 11] are key concepts in both mindfulness theory and mindfulness based clinical approaches, e.g. mindfulness-based cognitive therapy (MBCT) [12]. Mindfulness has been attempted to be operationalized with the Five Facet Mindfulness Questionnaire (FFMQ) [10], which measures different aspects of mindfulness. These aspects include being conscious of one’s actions instead of being distracted by thoughts, accepting inner experiences instead of judging them, and not reacting to inner experiences but letting them come and go. They also include observing, or paying attention to, sensory experiences, and being able to describe thoughts and feelings. MBCT [12] aims to combine mindfulness training with certain elements from cognitive therapy.

Acceptance and commitment therapy (ACT) [4] builds upon theory from behavior therapy, cognitive therapy, and mindfulness. Excessive or improper regulation of behavior by verbal processes is termed cognitive fusion in ACT [13]. Cognitive fusion may lead to psychological inflexibility causing people to act in ways that are inconsistent with their goals. The Acceptance and Action Questionnaire-II (AAQ-II) [14] operationalizes psychological flexibility and experiential avoidance. Experiential avoidance denotes an individual’s attempt to alter the form, frequency, or situational sensitivity of difficult mental events [14]. In ACT, it is believed that experiential avoidance creates and sustains psychological disorder, and that its opposite, psychological flexibility, gives opportunities for improved decision making and psychological well-being [4].

Symptoms of anxiety and depression often co-occur [15] and studies have found metacognition [16], mindfulness [17] and experiential avoidance [18] to be related to both anxiety and depression. However, few studies have explored similarities and differences between these theoretical constructs. One study [19] found metacognition and mindfulness to be separate constructs. Metacognition and mindfulness predicted symptoms of depression, but only metacognition predicted anxiety. However, experiential avoidance was not assessed in the study.

Although there are important differences between different third wave therapies, some theoretical concepts such as the attempt to change experiential awareness and the emphasis on acceptance share common ground. The primary aim of this study was to explore how metacognition, mindfulness, and experiential avoidance/psychological flexibility might relate to each other, as well as exploring the extent to which these constructs explain symptoms of anxiety and depression. The study has the following research questions:

1. What are similarities and differences between metacognition, mindfulness, and experiential avoidance/psychological flexibility?
2. What is the relative contribution of metacognition, mindfulness, and experiential avoidance/psychological flexibility when explaining symptoms of anxiety and depression?

## Methods

### Participants and procedure

A community sample of 364 people took part in an internet survey. The link to the survey was distributed on social media platforms like Facebook and all data were collected anonymously with no contact between the researchers and the respondents.  The survey program used allowed no missing values. Anyone over the age of 18 could participate. The age ranged between 18 and 76, with a mean age of 38.44 (*SD* = 13.19). The sample consisted of 100 men (27.5%) and 264 women (72.5%). Slightly more than a third of the sample (35.4%) had at one point in time been diagnosed with a mental illness. For more detailed information regarding the sample see Table 1. The project was approved by the Norwegian Centre for Research Data (reference number 340231).

**Table 1:** Summary of participant characteristics

	*n*	%
Female	264	72.5
Male	100	27.5
Single	117	32.1
In a relationship	43	11.8
Married/cohabiting	202	55.5
Full-time workers	224	61.5
Students	82	22.5
Unemployed	45	12.4
Retired	13	3.6
Currently or previously suffered from a mental disorder	129	35.4

### Measures

The Metacognitions Questionnaire-30 (MCQ-30) [6] is a 30-item questionnaire that measures metacognitions related to five subscales: *cognitive confidence*, *negative beliefs* (concerning the danger and uncontrollability of thoughts), *beliefs about the need to control thoughts*, *positive beliefs about worry* and *cognitive self-consciousness*. MCQ-30 uses a 4-point Likert scale from 1 to 4 and the total score ranges between 30 and 120. Higher scores indicate more dysfunctional metacognitions, and all subscales have positive relationships with pathological worry and trait anxiety [19]. MCQ-30 shows good reliability and convergent validity [6].

The Five Facet Mindfulness Questionnaire-24 (FFMQ-24) consists of 24 items using a 5-point Likert scale. FFMQ contains five subscales: *observing*, *describing*, *acting with awareness*, *non-judging of inner experience* and *non-reactivity to inner experience*. A higher score on FFMQ indicates that the respondent is more mindful. The Norwegian FFMQ has been validated for use in Norway [20]. FFMQ has been shown to have acceptable reliability and validity [21, 22]. The original FFMQ consists of 39 items, but a version with 24 items is considered valid and reliable [23]. The 24-item version was used in the current study.

The Acceptance and Action Questionnaire-II (AAQ-II) [14] is a 7-item scale that measures experiential avoidance and psychological inflexibility. AAQ-II uses a 7-point Likert scale and the total score of AAQ-II ranges between 0 and 49. Higher scores indicate less psychological flexibility and more experiential avoidance. AAQ-II is considered a valid and reliable measure of experiential avoidance (Bond et al., 2011).

The Generalized Anxiety Disorder 7-item (GAD-7) [24] contains seven items and uses a 4-point Likert scale. The total score, ranging from 0 to 21, indicates levels of anxiety. The questionnaire was developed to measure the core symptoms of generalized anxiety disorder but is also performs well as a measure of other types of anxiety [25]. A cut-off of 8 and 10 points has been suggested [24, 26]. The GAD-7 is considered to be a valid and reliable measure of anxiety, also in heterogeneous samples [26].

The Patient Health Questionnaire-9 (PHQ-9) [27] consists of nine items concerning symptoms of depression. The items are answered based on experiences in the last two weeks, on a 4-point Likert scale. The items build upon the nine criteria for depression in DSM-IV [28]. Suggested cut-off scores range between 8 and 13, but 10 is commonly used [29]. Studies indicate that the PHQ-9 has good reliability and validity [30].

### Statistics

Box plots revealed one respondent with extreme scores on all measures. This outlier was removed from the data set, leaving 363 respondents for the statistical analyses. Pearson correlation analyses were used to explore the relationships between metacognition, mindfulness, experiential avoidance, and symptoms of anxiety and depression. Two multiple regression analyses (model 1) were conducted using GAD-7 and PHQ-9 as dependent variables and AAQ-II, MCQ-30, and FFMQ-24 as predictor variables. For the correlation and regression analyses, Bonferroni corrections were conducted to account for multiple comparisons. A Maximum Likelihood factor analysis using direct obliminal rotation was used to further explore the relationships between metacognition, mindfulness, and experiential avoidance. For inclusion in a factor, variables needed a loading above 0.4, side loadings could not be above 0.3, and the difference between loadings had to be larger than 0.2. In order to investigate whether the extracted factors explained variance in symptoms of depression and anxiety, two hierarchical regression analyses were conducted (model 2) using the PHQ-9 and GAD-7 as dependent variables.

## Results

The mean score on PHQ-9 was 6.32 (*SD* = 4.83). Using a cut-off score of 10, 20.9% showed indications of depression. In this sample, 7.7% reported that their problems with depression made it “very difficult” to function in everyday life, and 1.4% as “extremely difficult”. The mean score on GAD-7 was 4.66 (*SD* = 3.95). Using a cut-off score of 8, 19.3% showed indications of anxiety. In this sample, 6.9% stated that their anxiety made everyday functioning “very difficult”, and 0.8% as “extremely difficult”. Table 2 gives an overview of the means, standard deviations, and internal consistencies of all included measures.

**Table 2:** Levels of metacognition, mindfulness, experiential avoidance, and symptoms (n = 363)

	Range	Min-Max	Mean	SD	α
MCQ-30	30–120	30–96	50.37	11.35	0.89
*Cognitive confidence*	6–24	6–24	9.80	3.51	0.84
*Positive beliefs about worry*	6–24	6–22	8.97	2.81	0.82
*Negative beliefs about worry*	6–24	6–24	10.89	3.84	0.81
*Cognitive self-consciousness*	6–24	6–24	12.44	3.77	0.80
*Need to control thoughts*	6–24	6–20	8.26	2.65	0.73
FFMQ-24	24–120	47–116	86.85	12.58	0.89
*Observing*	4–20	4–20	15.15	3.21	0.80
*Describing*	5–25	8–25	19.77	3.66	0.86
*Acting with awareness*	5–25	6–25	17.18	3.45	0.81
*Non-reactivity to inner experience*	5–25	7–25	16.82	3.76	0.82
*Non-judging of inner experience*	5–25	6–25	17.93	4.50	0.80
AAQ-II	7–49	7–46	18.50	8.73	0.92
PHQ-9	0–27	0–25	6.32	4.83	0.87
GAD-7	0–21	0–19	4.66	3.95	0.88

*Note*. MCQ-30 = Metacognitions Questionnaire-30; FFMQ-24 = Five Facet Mindfulness Questionnaire-24; AAQ-II = Acceptance & Action Questionnaire-II; PHQ-9 = Patient Health Questionnaire-9; GAD-7 = Generalized Anxiety Disorder 7-item

All FFMQ-24 subscales correlated negatively and significantly with AAQ-II, except for *observing*. The FFMQ-24 subscales and the MCQ-30 total score also correlated negatively and significantly, except for non-reactivity to inner experiences with cognitive confidence. *Non-judging of inner experience* showed the strongest correlations with AAQ-II and MCQ-30 (-0.66 and − 0.68, respectively). The AAQ-II and the MCQ-30 showed a moderate-strong correlation (0.67). The FFMQ-24 subscales *non-judging of inner experience* and *non-reactivity to inner experience* showed the strongest correlations with the MCQ-30. All subscales of the MCQ-30 had positive and significant correlations with both PHQ-9 (ranging between 0.28 and 0.53) and GAD-7 (ranging between 0.21 and 0.57). AAQ-II had a positive and significant correlation with PHQ-9 (0.71) and GAD-7 (0.64). The correlations between FFMQ-24 and PHQ-9 were moderate to strong (both − 0.57). An overview of the correlational analyses is presented in Table 3.

**Table 3:** Correlations between MCQ-30 subscales, FFMQ-24 subscales, AAQ-II, and symptom measures (n = 363)

	2	3	4	5	6	7	8	9	10	11	12	13	14	15
1 GAD-7	0.72	0.64	0.21	0.44	0.57	0.41	0.41	0.60	− 0.05	− 0.35	− 0.43	− 0.55	− 0.48	− 0.57
2 PHQ-9		0.71	0.28	0.40	0.53	0.29	0.43	0.57	− 0.08	− 0.33	− 0.44	− 0.55	− 0.46	− 0.57
3 AAQ-II			0.27	0.47	0.65	0.36	0.52	0.67	− 0.05	− 0.40	− 0.47	− 0.66	− 0.58	− 0.67
4 Cognitive confidence				0.16	0.20	0.09	0.21	0.50	− 0.07	− 0.26	− 0.34	− 0.21	− 0.15	− 0.30
5 Cognitive self-consciousness					0.48	0.35	0.46	0.72	0.06	− 0.16	− 0.26	− 0.52	− 0.28	− 0.37
6 Negative beliefs						0.41	0.59	0.80	− 0.07	− 0.31	− 0.39	− 0.63	− 0.51	− 0.59
7 Positive beliefs							0.41	0.63	− 0.06	− 0.21	− 0.18	− 0.37	− 0.29	− 0.34
8 Need to control thoughts								0.75	− 0.03	− 0.29	− 0.27	− 0.62	− 0.33	− 0.48
9 MCQ-30 total score									− 0.05	− 0.36	− 0.43	− 0.68	− 0.46	− 0.62
10 Observing										0.25	0.18	0.06	0.31	0.49
11 Describing											0.42	0.33	0.41	0.71
12 Acting with awareness												0.41	0.33	0.70
13 Non-judging of inner exp.													0.45	0.72
14 Non-reactivity to inner exp.														0.75
15 FFMQ total														

*Note*. All correlations at or above r = .21 were significant following Bonferroni correction (*p* < .0005).” GAD-7 = Generalized Anxiety Disorder 7-item; PHQ-9 = Patient Health Questionnaire-9; AAQ-II = Acceptance and Action Questionnaire-II; MCQ-30 = Metacognitions Questionnaire-30; FFMQ-24 = Five Facet Mindfulness Questionnaire-24

The maximum likelihood factor analysis extracted two factors. Factor 1 was dominated by MCQ-30 subscales and appeared to represent a *flexibility in cognition and emotional experience* factor. Factor 2 was dominated by FFMQ-24 subscales (*describing*, *acting with awareness*, and *observing*) and appeared to reflect *present-centered attention and awareness*. The AAQ-II and the FFMQ-24 subscale *non-judging of inner experience* loaded onto the flexibility factor. The subscales MCQ Cognitive confidence and FFMQ Non-reactivity to internal experience did not meet the criteria for inclusion in a factor and were excluded. To explore the associations between the two factors that emerged in factor analysis and the symptom measures, zero-order and partial correlations (controlling for the extracted factors) were used and showed that the flexibility factor had stronger correlations with symptoms than the present-centered factor. Table 4 summarizes the results of the factor analysis and the correlations between factors and symptoms.

**Table 4:** Maximum Likelihood factor analysis and correlations between factors and symptom measures

	Factor 1 Flexibility		Factor 2 Present-centered attention/awareness		
FFMQ *Non-judging*	**− 0.79**		0.08		
MCQ *Negative Beliefs*	**0.78**		− 0.08		
MCQ *Need to control thoughts*	**0.75**		0.05		
AAQ-II	**0.71**		− 0.23		
MCQ *Cognitive self-consciousness*	**0.69**		0.12		
MCQ *Positive beliefs*	**0.51**		0.02		
FFMQ *Describing*	− 0.20		**0.56**		
FFMQ *Acting with awareness*	− 0.29		**0.52**		
FFMQ *Observing*	0.10		**0.40**		
		Correlations			
	Zero-order	Partial	Zero-order		Partial
PHQ-9	0.58*	0.26*	− 0.39*		− 0.18*
GAD-7	0.61*	0.35*	− 0.38*		− 0.16*
Flexibility			− 0.34*		

*Note.* *significant following Bonferroni correction (*p* < .005). MCQ *Cognitive confidence* and *FFMQ Non-reactivity to internal experience* were not included in the table due to the subscales’ low loadings (< 0.4) on both factors. Variables needed a loading above 0.4, side loadings could not be above 0.3, and the difference between loadings had to be larger than 0.2. MCQ = Metacognitions Questionnaire; FFMQ = Five Facet Mindfulness Questionnaire; AAQ-II = Acceptance and Action Questionnaire-II = AAQ-II; PHQ-9 = Patient Health Questionnaire-9; GAD-7 = Generalized Anxiety Disorder 7-item

Two regression analyses (model 1) investigated variables associated with symptoms of depression and anxiety. Female sex, AAQ-II, MCQ-30, and FFMQ-24 were significantly associated with symptoms of anxiety, explaining 49% of the variance. Being out of work, AAQ-II, and FFMQ-24 were significantly associated with symptoms of depression, explaining 56% of the variance. A summary of the regression analyses is presented in Table 5.

**Table 5:** Hierarchical regression analysis with GAD-7 and PHQ-9 as dependent variables (n = 363)

	*GAD-7 (*Adj. *R*^2^ = 0.49)			*PHQ-9 (*Adj. *R*^2^ = 0.56)		
*Model 1*	*β*	*t*	*p*	*β*	*t*	*p*
Sex	0.107	2.812	0.005*	0.021	0.593	0.553
Age	0.004	0.106	0.916	− 0.020	-0.543	0.588
Education	− 0.018	-0.456	0.649	− 0.086	-2.315	0.021
Out of work	0.009	0.231	0.818	− 0.154	-4.098	< 0.001*
Mental health history	0.073	1.756	0.080	0.088	2.281	0.023
AAQ-II	0.302	5.004	< 0.001*	0.436	7.785	< 0.001*
MCQ-30	0.260	4.804	< 0.001*	0.108	2.144	0.033
FFMQ-24	− 0.197	-3.615	< 0.001*	− 0.158	-3.129	0.002*
*Model 2*	Adj. *R*^2^	*R*^2^ ∆	Sign. *F* ∆	Adj. *R*^2^	*R*^2^ ∆	Sign. *F* ∆
1. Demographics	0.12	0.13	< 0.001**	0.23	0.24	< 0.001**
2. Present-centered	0.24	0.12	< 0.001**	0.34	0.11	< 0.001**
3. Flexibility	0.44	0.20	< 0.001**	0.47	0.12	< 0.001**
Final step	*β*	*t*	*P*	*β*	*t*	*p*
Sex	0.141	3.532	< 0.001*	0.063	1.608	0.109
Age	− 0.005	-0.123	0.902	− 0.032	-0.802	0.423
Education	− 0.040	-0.950	0.343	− 0.099	-2.429	0.016
Out of work	− 0.009	-0.218	0.827	− 0.192	-4.669	< 0.001*
Mental health history	0.126	3.020	0.003*	0.171	4.165	< 0.001*
Present-centered	− 0.201	-4.719	< 0.001*	− 0.224	-5.384	< 0.001*
Flexibility	0.507	11.439	< 0.001*	0.395	9.123	< 0.001*

*Note.* *significant following Bonferroni correction (*p* < .007). GAD-7 = Generalized Anxiety Disorder 7-item; AAQ-II = Acceptance & Action Questionnaire-II; MCQ-30 = Metacognitions Questionnaire-30; FFMQ-24 = Five Facet Mindfulness Questionnaire-24. The largest VIF values obtained were 2.58 for model 1, and 1.27 for model 2, suggesting no serious concerns with multicollinearity

Another two regression analyses (model 2) used the present-centered and flexibility factors as independent variables. Demographic variables were entered in the first step, present-centered attention/awareness in step 2, and the flexibility factor in the third and final step. The results showed that female sex, mental health history, present-centered attention/awareness, and flexibility were significantly associated with symptoms of anxiety. The model explained 44% of the variance in symptoms, and the flexibility factor showed the strongest beta value (0.51). Being out of work, having a history of mental health problems, present-centered attention/awareness, and flexibility were significantly associated with symptoms of depression. The model explained 47% of the variance in symptoms, and again flexibility showed the largest beta value (0.40). A summary of the hierarchical regression analysis related to the factors is presented in Table 5.

## Discussion

The study found that there are significant associations between metacognition, mindfulness, and experiential avoidance, and that all were associated with symptoms of anxiety and depression. The factor analysis suggested two separate factors; a flexibility in cognition and emotional experience factor constituted of metacognition (MCQ-30), experiential avoidance (AAQ-II), and *non-judging* (from the FFMQ), and a present-centered attention and awareness factor (consisting of FFMQ subscales). Both factors were significantly associated with symptoms, but the flexibility factor showed a stronger relationship with both anxiety and depression. This replicated the findings of Solem et al. [19] and extends it by including the AAQ-II, thus supporting the notion that metacognitions and how a person relates to cognition and emotional experiences are important in the development of symptoms of anxiety and depression. The identification of a present moment attention and awareness factor is in line with Brown and Ryan’s [31] definition of mindfulness “as an *open* or *receptive* attention to and awareness of ongoing events and experience” (p. 245).

*Non-judging of inner experience* loaded negatively onto the flexibility factor, which could indicate that judging one’s thoughts and feelings is more associated with healthy regulation of thought and emotion than other mindfulness variables. From a metacognitive perspective, the reason why this item loaded more strongly on the flexibility factor than the present-centered attention and awareness factor, could be that psychological symptoms are closely related to extended thinking [5]. Judging one’s inner experiences could be related to activation of the CAS, which again may lead to symptoms of anxiety or depression and sustain pre-existing symptoms.

The FFMQ measures mindfulness as a multifaceted construct, and it is worth noting that three of the five facets loaded on the present-centered attention and awareness factor. The results of this study therefore suggest that mindfulness as operationalized with the FFMQ may involve two concepts that are distinct but related, and which may have different associations with anxiety and depression. The FFMQ subscale *non-reactivity to internal experience* did not have a clear loading onto any of the extracted factors. However, previous research [19] found non-reactivity to load onto a mindfulness factor. The moderate to strong correlation between the non-reactivity subscale and symptoms of anxiety and depression suggests that this facet may still be important for mental health. In clinical practice, dealing with disturbing thoughts and emotions in a non-reactive manner could be helpful.

The AAQ-II loaded onto the flexibility factor, which implies that experiential avoidance could reflect strategies for regulation cognition and emotion. Some research suggests that the CAS significantly overlaps with experiential avoidance [32]. One aspect of the CAS is unhelpful coping behaviors, which includes avoidance of thoughts as opposed to letting thoughts go [5], thus this may be understood as experiential avoidance. Engaging in experiential avoidance can also predict the development of negative metacognitive beliefs [32]. Metacognitive theory suggests that negative meta-beliefs are closely related to the CAS, which can interfere with healthy adaptation to challenging emotional experiences and thereby making symptoms persist. Relatedly, ACT theory states that experiential avoidance gets in the way of a psychologically more flexible approach to dealing with challenging emotional experiences, which in turn causes or sustains symptoms [13]. Thus, there appears to be some similarity between these constructs with regards to avoidance.

We also investigated the relation between the present-centered and flexibility factors and symptoms of anxiety and depression in separate regression analyses. The analyses showed that the flexibility factor was more strongly related to both types of symptoms compared to the present-centered factor. Both the MCQ-30 items and the AAQ-II loaded on the flexibility factor, and the regression analyses showed a strong association between flexibility and anxiety and depression symptoms. However, there was a moderate correlation between the two factors identified, which indicates some overlap between the factors even though they were related to psychological symptoms to different extents. These results suggest that although there are similarities between theoretical constructs in different third wave therapies, there may also be important differences. It may be that the flexibility factor measures maladaptive cognitive dysfunction and emotional dysregulation related to psychological problems to a larger extent than mindfulness. These results mirror those of Solem et al. [19] and lend further support to the metacognitive model of psychological vulnerability. The results of the regression analyses must be treated with some caution as we used the total score of the FFMQ, which has been advised against by the originators of the scale [10]. However, a meta-analysis [17] concluded that it may be useful to consider trait mindfulness both multi-dimensionally and as a unitary construct. Although clinical implications must be considered tentative, the results may imply that emphasis should be devoted to improving flexibility in cognitive and emotional regulation skills rather than targeting present-moment attention and awareness in clinical work. Future studies should explore whether targeting specifically metacognitions could be a more effective treatment intervention, as suggested by preliminary evidence [33].

Although the results of the present study add to the empirical literature on metacognition, mindfulness, and experiential avoidance, the study has some limitations which must be noted. The data in the study was cross-sectional, meaning that inferences about causality cannot be made. Future studies should seek to use a repeated measures design. The data was also based on self-report measures. Self-report measures may entail disturbances to the data caused by, among other things, social desirability, selective memory, and selective or subjective interpretations of the items in the measures. Furthermore, the convenience sampling used in this study is a limitation, and therefore the study should be replicated in a clinical sample.

## Conclusions

Overall, the study showed that although metacognition, mindfulness and experiential avoidance have certain similarities, the factor reflecting flexibility in cognition and emotional experience was more associated with mental health symptoms. The findings suggest that metacognitions, experiential avoidance and non-judging of inner experiences, could be important in explaining symptoms of anxiety and depression.

## Data Availability

The datasets used in the current study are available from the corresponding author on reasonable request.

## List of abbreviations

AAQ-II: Acceptance and Action Questionnaire-II
ACT: Acceptance and Commitment Therapy
BT: Behavior Therapy
CAS: Cognitive Attentional Syndrome
CBT: Cognitive Behavior Therapy
CT: Cognitive Therapy
DSM-IV: Diagnostic and statistical manual of mental disorders (4th ed)
FFMQ-24: Five Facet Mindfulness Questionnaire-24
GAD-7: Generalized Anxiety Disorder 7-item
MBCT: Mindfulness Based Cognitive Therapy
MCT: Metacognitive Therapy
MCQ-30: Metacognitions Questionnaire-30
PHQ-9: Patient Health Questionnaire 9-item
