# Correction: An empirical investigation of the associations between metacognition, mindfulness experiential avoidance, depression, and anxiety

**DOI:** 10.1186/s40359-023-01394-x

**Published:** 2023-10-23

**Authors:** Torstein Ådnøy, Stian Solem, Roger Hagen, Audun Havnen

**Affiliations:** 1 Department of Psychology, Norwegian University of Science and Technology, Trondheim, Norway; 2 Department of Psychology, University of Oslo, Oslo, Norway; 3 Research institute, Modum Bad, Vikersund, Norway; 4 Division of Psychiatry, Nidaros Community Mental Health Centre, St. Olavs University Hospital, Trondheim, Norway

**BMC Psychology (2023) 11:281**

10.1186/s40359-023-01336-7

Following publication of the original article [1], the authors flagged the following error in the Results section of the Abstract: where it now says ‘Metacognition, experiential avoidance, and the non-judging subscale of FFMQ-24 constituted’, ‘Mindfulness’ had erroneously been written in place of ‘Metacognition’. The published article has since been corrected. The authors thank you for reading this erratum and apologize for any inconvenience caused.

